# Supplementary material for: Caspase-1 activates gasdermin A in non-mammals
Source: bioRxiv. 2024 Jan 3:2023.09.28.559989. Originally published 2023 Sep 28. Preprint. [Version 3] doi: 10.1101/2023.09.28.559989 (PMC10659411; doi:10.1101/2023.09.28.559989)
Supplement: Supplement 1 [file NIHPP2023.09.28.559989v3-supplement-1.pdf]

bioRxiv preprint doi: https://doi.org/10.1101/2023.09.28.559989; this version posted January 3, 2024. The copyright holder for this preprint (which was not certified by peer review) is the author/funder. This article is a US Government work. It is not subject to copyright under 17 USC 105 and is also made available for use under a CC0 license.

Supplementary Data Files.

- Figure 1—source data 1. Tree in .tre format.
- Figure 1—source data 2. Alignment file of all sequences in nexus format.
- Figure 1—figure supplement 1—source data 1. Text file with all sequences with seqID and ID used in nexus alignment file.
- Figure 1—figure supplement 3—source data 1. Maximum likelihood tree in .nwk format.
- Figure 1—figure supplement 3—source data 2. Maximum likelihood tree with bootstrap values.
- Figure 1—figure supplement 3—source data 3. Maximum likelihood tree with node tips labeled.
- Figure 1—figure supplement 3—source data 4. Maximum likelihood tree with node tips labeled and bootstrap values.
- Figure 5—source data 1. AlphaFold prediction files in .pdb format.

bioRxiv preprint doi: https://doi.org/10.1101/2023.09.28.559989; this version posted January 3, 2024. The copyright holder for this preprint (which was not certified by peer review) is the author/funder. This article is a US Government work. It is not subject to copyright under 17 USC 105 and is also made available for use under a CC0 license.

871 ## Supplemental Figures:

872 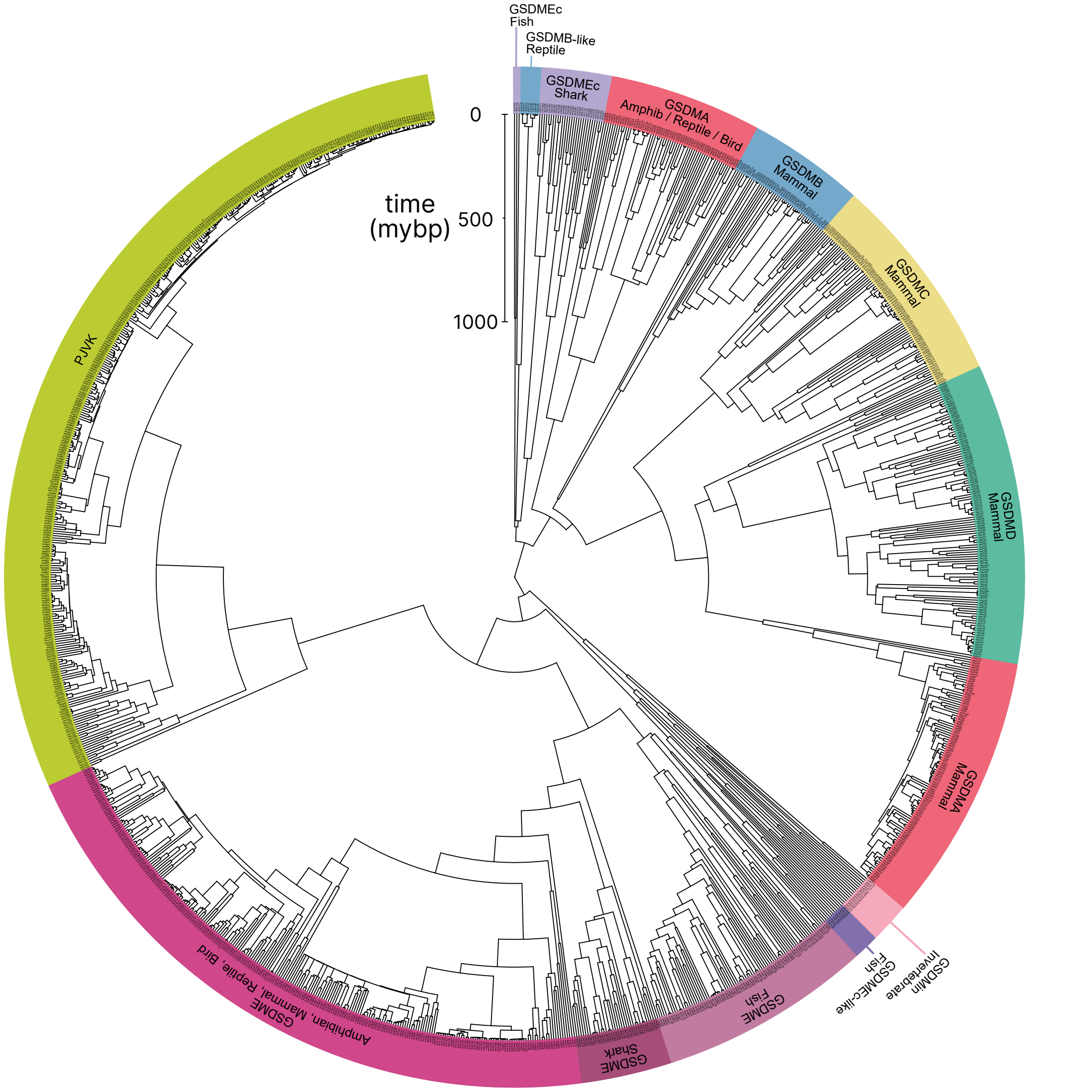

Figure 1—figure supplement 1. Tree identical to Figure 1 with individual nodes labeled.

Figure 1—figure supplement 1—source data 1. Text file with all sequences with seqID and ID used in nexus alignment file.

bioRxiv preprint doi: https://doi.org/10.1101/2023.09.28.559989; this version posted January 3, 2024. The copyright holder for this preprint (which was not certified by peer review) is the author/funder. This article is a US Government work. It is not subject to copyright under 17 USC 105 and is also made available for use under a CC0 license.

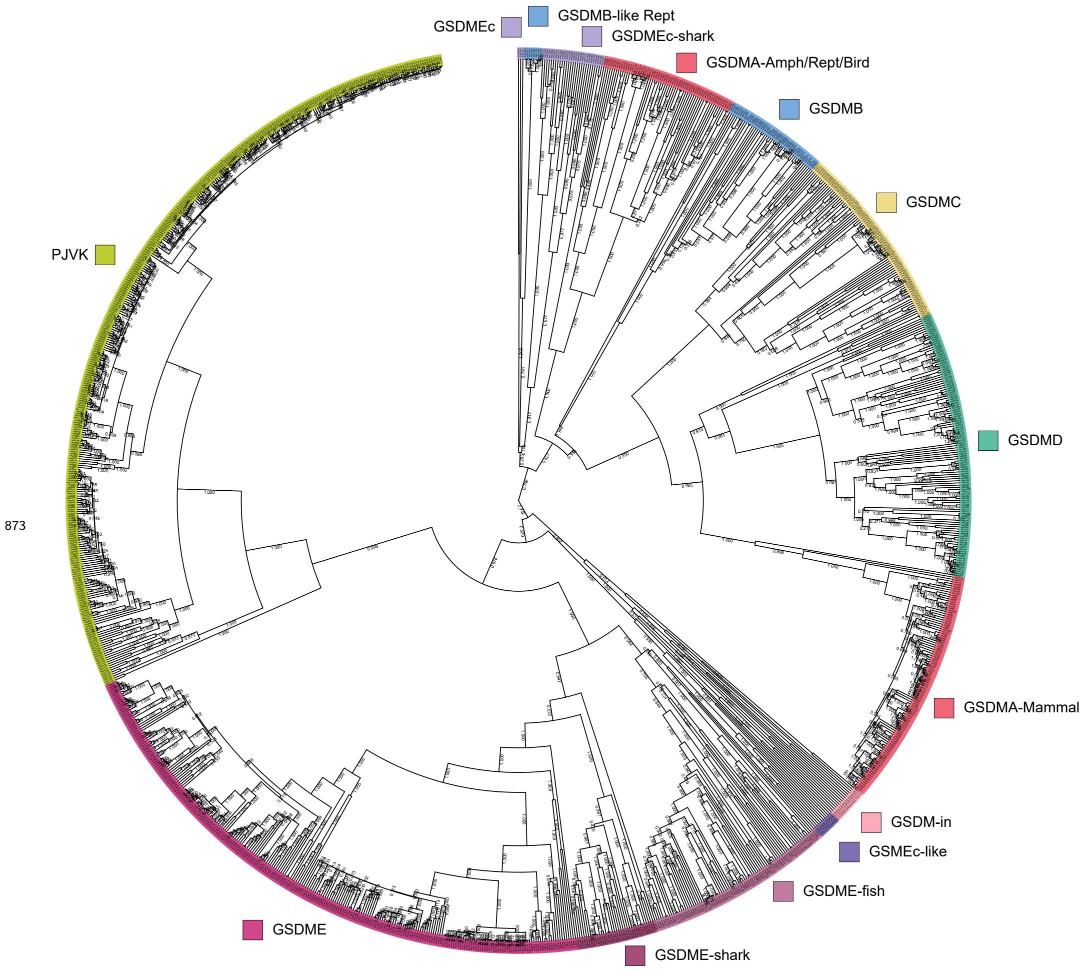

Figure 1—figure supplement 2. Tree identical to Figure 1 with individual nodes labeled and posterior probabilities displayed at the midpoint of each node.

bioRxiv preprint doi: https://doi.org/10.1101/2023.09.28.559989; this version posted January 3, 2024. The copyright holder for this preprint (which was not certified by peer review) is the author/funder. This article is a US Government work. It is not subject to copyright under 17 USC 105 and is also made available for use under a CC0 license.

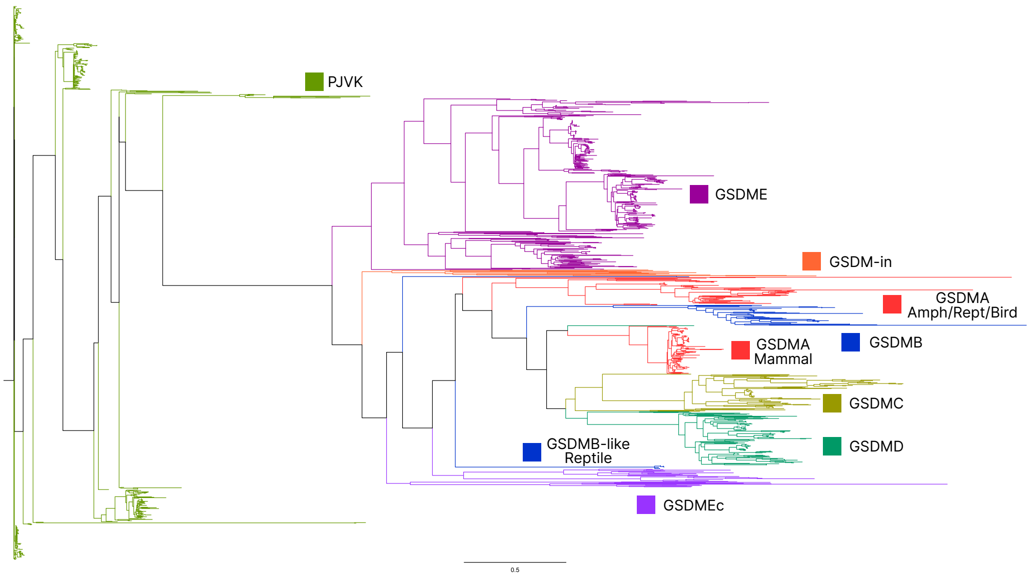

Figure 1—figure supplement 3. Maximum likelihood tree generated using the same sequences in Figure 1 Bayesian tree.
Figure 1—figure supplement 3—source data 1. Maximum likelihood tree in Newick format.
Figure 1—figure supplement 3—source data 2. Maximum likelihood tree with bootstrap values.
Figure 1—figure supplement 3—source data 3. Maximum likelihood tree with node tips labeled.
Figure 1—figure supplement 3—source data 4. Maximum likelihood tree with node tips labeled and bootstrap values.

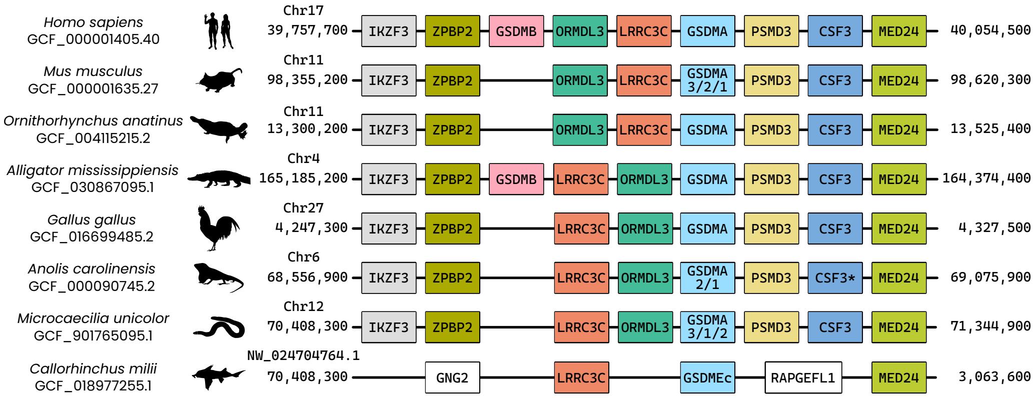

Figure 1—figure supplement 4. Visualization of the locus where *GSDMA* is found in the clades of animals that encode this gasdermin. In tetrapods, the genes found in this region are identical, though *ORMDL3* and *LRRC3C* are in opposing positions in some animals. Note that *Microcaecilia unicolor* (Tiny Cayenne caecilian) is an amphibian despite appearing morphologically similar to a snake. Duplications of *GSDMA* are indicated by numbers below *GSDMA*. Though not annotated as *CSF3* in *Anolis carolinensis* (Carolina anole), the protein encoded by this sequence aligns with G-CSF (encoded by *CSF3*) in mammals by BLASTp. It is colored and labeled as *CSF3* here for ease of appreciating synteny. In fish that encode *GSDMEc*, like *Callorhinchus milii* (elephant shark), *GSDMEc* is found in a similar locus near *LRRC3Ca* and *MED24*.

bioRxiv preprint doi: https://doi.org/10.1101/2023.09.28.559989; this version posted January 3, 2024. The copyright holder for this preprint (which was not certified by peer review) is the author/funder. This article is a US Government work. It is not subject to copyright under 17 USC 105 and is also made available for use under a CC0 license.

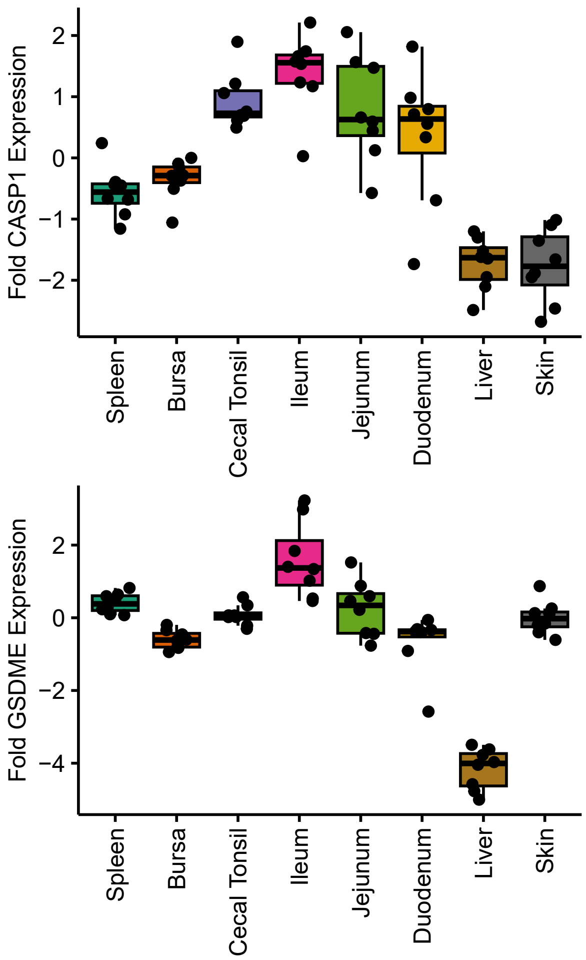

Figure 3—figure supplement 1. The same tissues as in Figure 3 were assayed for *GSDME* and *CASP1* alongside *GSDMA*.

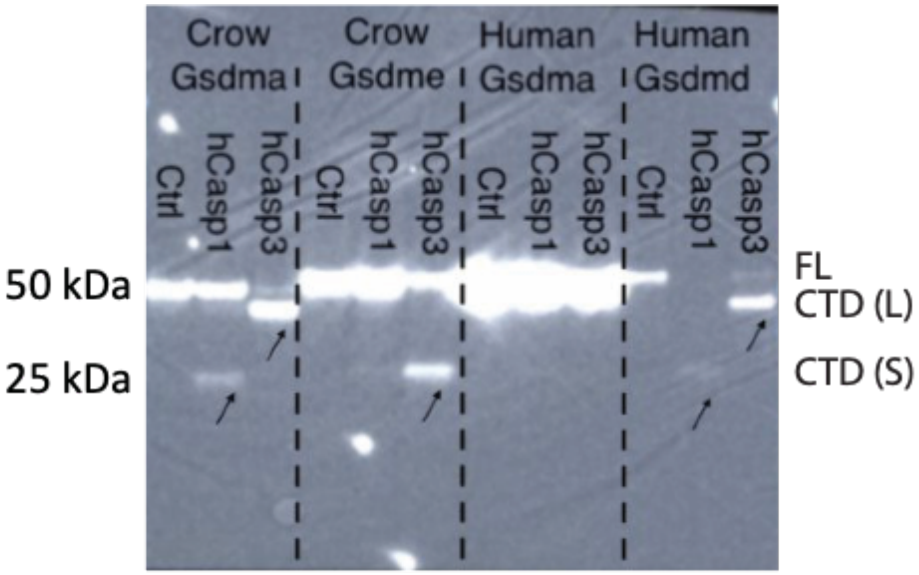

Figure 3—figure supplement 2. Incubation of 293T/17 lysates transfected with C-terminally tagged crow GSDMA, crow GSDME, human GSDMA, or human GSDMD then were incubated with human CASP1 or CASP3.

bioRxiv preprint doi: https://doi.org/10.1101/2023.09.28.559989; this version posted January 3, 2024. The copyright holder for this preprint (which was not certified by peer review) is the author/funder. This article is a US Government work. It is not subject to copyright under 17 USC 105 and is also made available for use under a CC0 license.

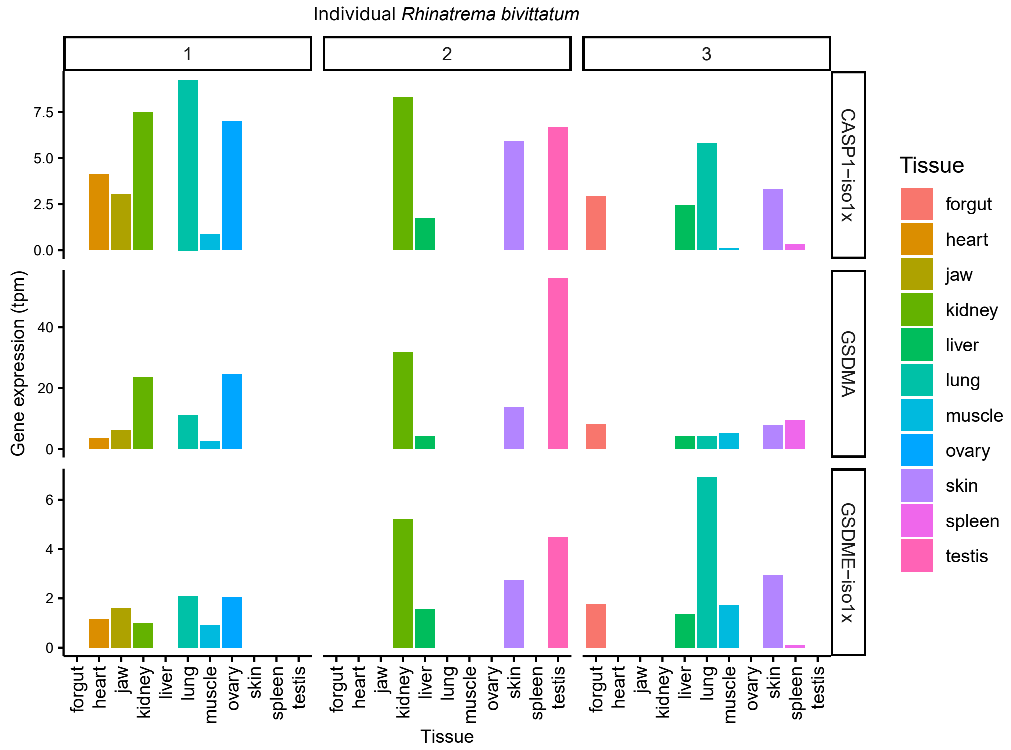

Figure 6—figure supplement 1. Amphibian expression of *CASP1*, *GSDMA* and *GSDME*. Not all amphibians had RNA extracted from the same organs. Note that kidney, when isolated, always has the highest expression of *GSDMA* of non-gonadal tissue.
