# Supplementary material for: Caspase-1 activates gasdermin A in non-mammals
Source: bioRxiv. 2023 Nov 20:2023.09.28.559989. Originally published 2023 Sep 28. Preprint. [Version 2] doi: 10.1101/2023.09.28.559989 (PMC10659411; doi:10.1101/2023.09.28.559989)
Supplement: Supplementary file 1 [file NIHPP2023.09.28.559989v2-supplement-1.pdf]

bioRxiv preprint doi: https://doi.org/10.1101/2023.09.28.559989; this version posted November 20, 2023. The copyright holder for this preprint (which was not certified by peer review) is the author/funder. This article is a US Government work. It is not subject to copyright under 17 USC 105 and is also made available for use under a CC0 license.

Supplementary Data Files.

- Figure 1—source data 1. Tree in .tre format.
- Figure 1—source data 2. Alignment file of all sequences in nexus format.
- Figure 1—figure supplement 1—source data 1. Text file with all sequences with seqID and ID used in nexus alignment file.
- Figure 1—figure supplement 3—source data 1. Maximum likelihood tree in .nwk format.
- Figure 1—figure supplement 3—source data 2. Maximum likelihood tree with bootstrap values.
- Figure 1—figure supplement 3—source data 3. Maximum likelihood tree with node tips labeled.
- Figure 1—figure supplement 3—source data 4. Maximum likelihood tree with node tips labeled and bootstrap values.
- Figure 5—source data 1. AlphaFold prediction files in .pdb format.

bioRxiv preprint doi: https://doi.org/10.1101/2023.09.28.559989; this version posted November 20, 2023. The copyright holder for this preprint (which was not certified by peer review) is the author/funder. This article is a US Government work. It is not subject to copyright under 17 USC 105 and is also made available for use under a CC0 license.

Supplemental Figures:

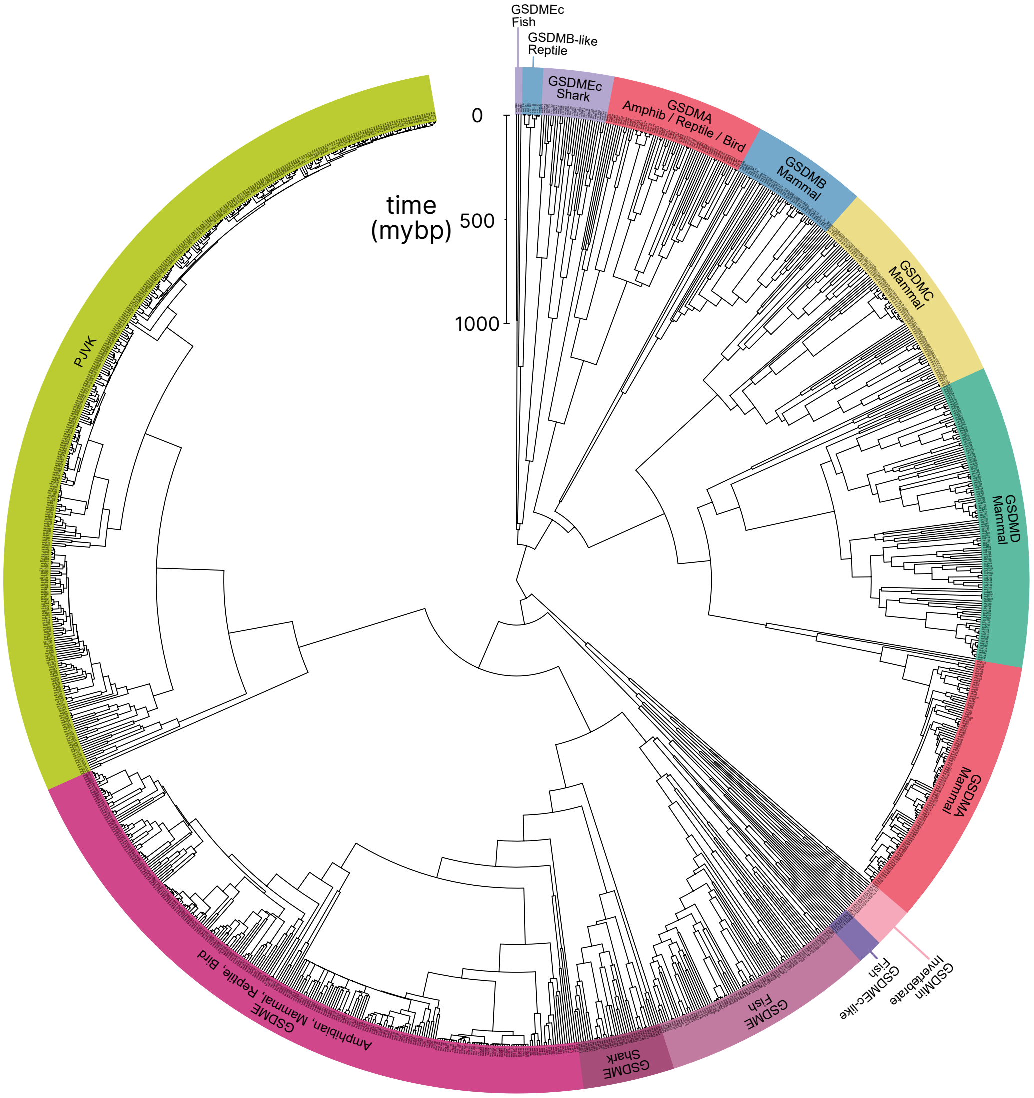

Figure 1—figure supplement 1. Tree identical to Figure 1 with individual nodes labeled.

Figure 1—figure supplement 1—source data 1. Text file with all sequences with seqID and ID used in nexus alignment file.

bioRxiv preprint doi: https://doi.org/10.1101/2023.09.28.559989; this version posted November 20, 2023. The copyright holder for this preprint (which was not certified by peer review) is the author/funder. This article is a US Government work. It is not subject to copyright under 17 USC 105 and is also made available for use under a CC0 license.

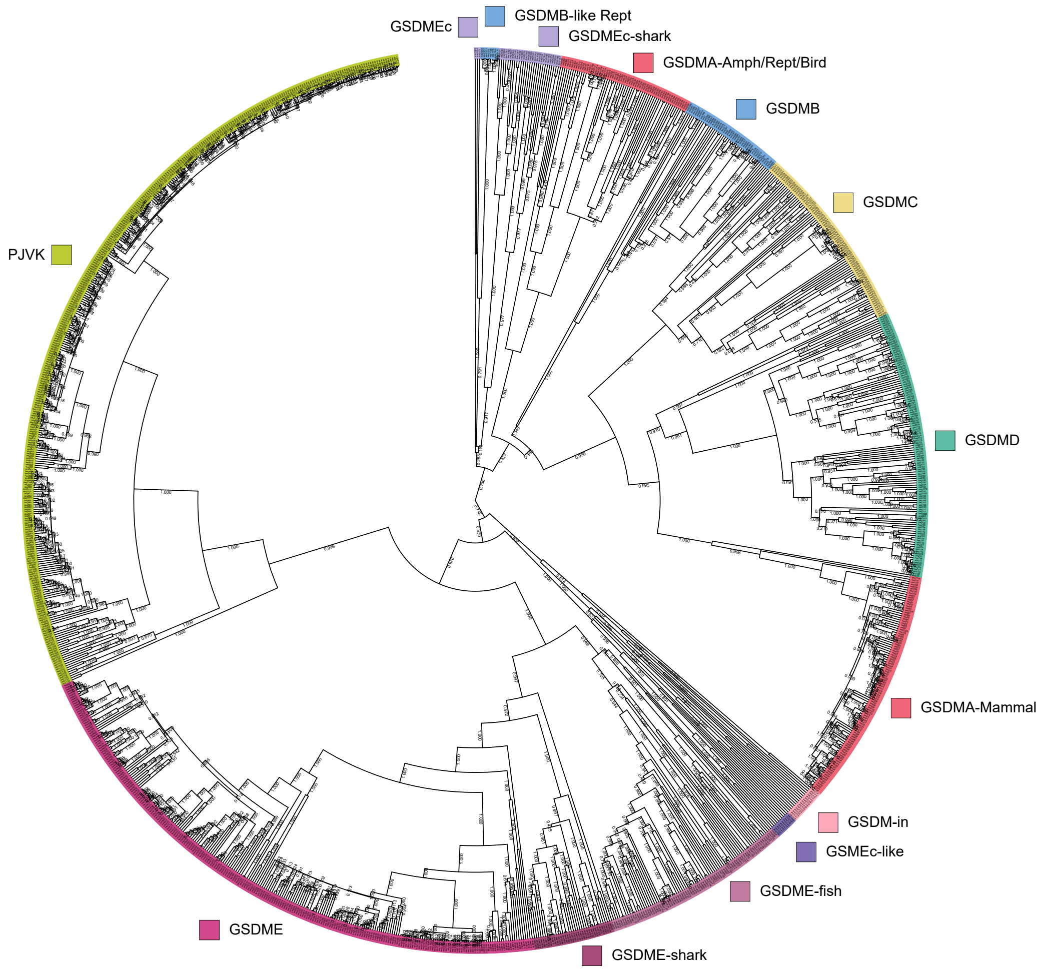

Figure 1—figure supplement 2. Tree identical to Figure 1 with individual nodes labeled and posterior probabilities displayed at the midpoint of each node.

bioRxiv preprint doi: https://doi.org/10.1101/2023.09.28.559989; this version posted November 20, 2023. The copyright holder for this preprint (which was not certified by peer review) is the author/funder. This article is a US Government work. It is not subject to copyright under 17 USC 105 and is also made available for use under a CC0 license.

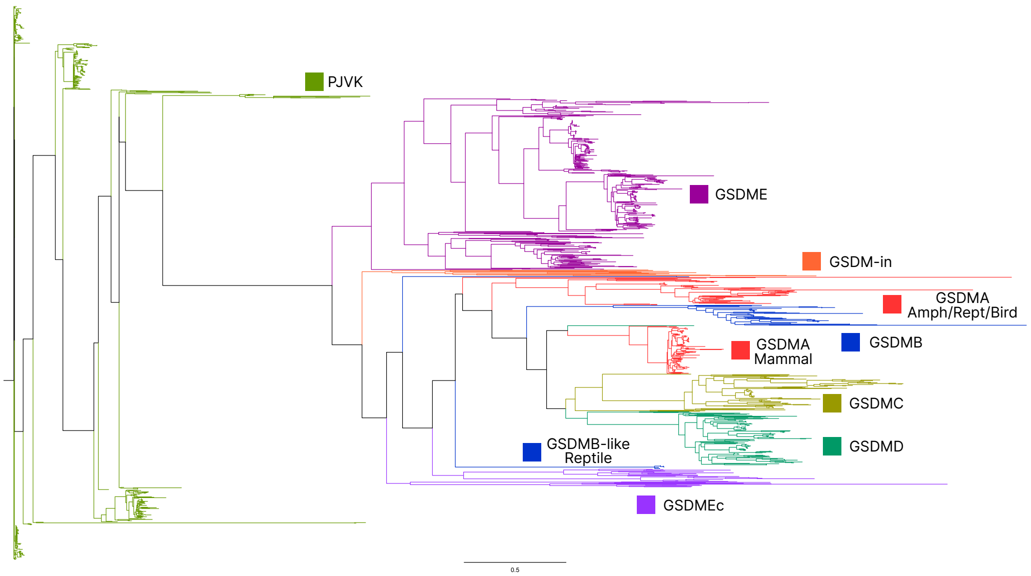

Figure 1—figure supplement 3. Maximum likelihood tree generated using the same sequences in Figure 1 Bayesian tree.

Figure 1—figure supplement 3—source data 1. Maximum likelihood tree in .nwk format.

Figure 1—figure supplement 3—source data 2. Maximum likelihood tree with bootstrap values.

Figure 1—figure supplement 3—source data 3. Maximum likelihood tree with node tips labeled.

Figure 1—figure supplement 3—source data 4. Maximum likelihood tree with node tips labeled and bootstrap values.

bioRxiv preprint doi: https://doi.org/10.1101/2023.09.28.559989; this version posted November 20, 2023. The copyright holder for this preprint (which was not certified by peer review) is the author/funder. This article is a US Government work. It is not subject to copyright under 17 USC 105 and is also made available for use under a CC0 license.

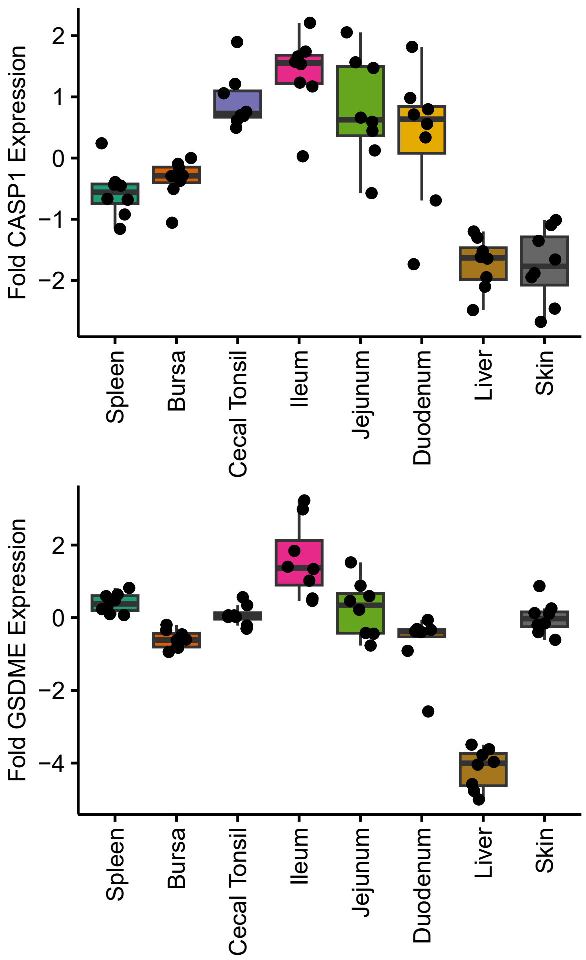

Figure 3—figure supplement 1. The same tissues as in Figure 3 were assayed for *GSDME* and *CASP1* alongside *GSDMA*.

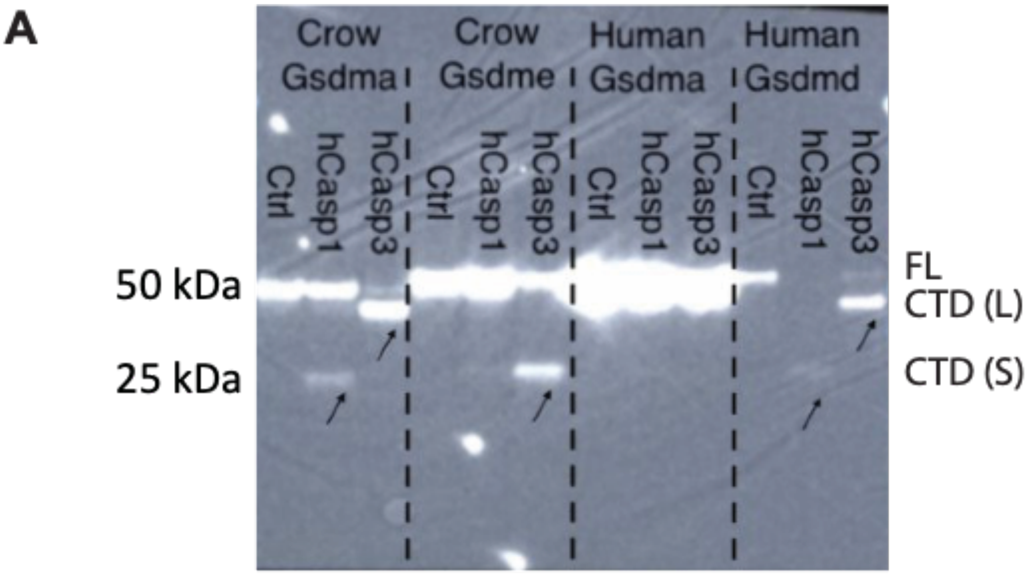

Figure 3—figure supplement 2. Incubation of 293T/17 lysates transfected with C-terminally tagged crow GSDMA, crow GSDME, human GSDMA, or human GSDMD then were incubated with human CASP1 or CASP3.

bioRxiv preprint doi: https://doi.org/10.1101/2023.09.28.559989; this version posted November 20, 2023. The copyright holder for this preprint (which was not certified by peer review) is the author/funder. This article is a US Government work. It is not subject to copyright under 17 USC 105 and is also made available for use under a CC0 license.

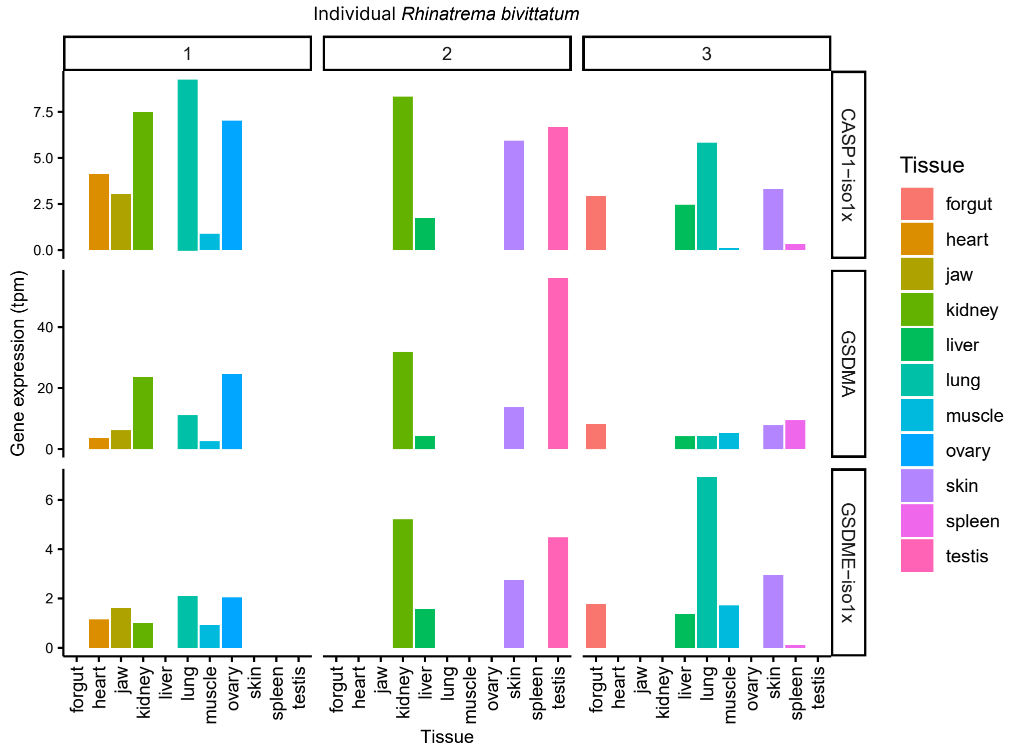

Figure 6—figure supplement 1. Amphibian expression of *CASP1*, *GSDMA* and *GSDME*. Not all amphibians had RNA extracted from the same organs. Note that kidney, when isolated, always has the highest expression of *GSDMA* of non-gonadal tissue.
